# 16S rRNA Gene Diversity of Bacterial Endophytes in Parasitic *Cuscuta campestris* and Its *Helianthus annuus* Host

**DOI:** 10.1128/MRA.00968-20

**Published:** 2020-10-22

**Authors:** Adam T. Avila, Tricia A. Van Laar, John V. H. Constable, Katherine Waselkov

**Affiliations:** a Department of Biology, California State University, Fresno, Fresno, California, USA; Indiana University, Bloomington

## Abstract

Here, we report the results of 16S rRNA gene amplicon sequencing of bacterial endophytes from parasitized and unparasitized samples of the common sunflower (*Helianthus annuus*) and samples of its associated plant parasite field dodder (*Cuscuta campestris*), collected from one location in Fresno County, California (August 2017).

## ANNOUNCEMENT

Bacterial endophytes (bacteria internal to the plant body) impact host plant growth, physiology, and disease (1, 2). Different species of plants and even individuals in the same plant population can have different endophyte communities (3–5). Plant parasites use modified root structures (haustoria) to penetrate host vascular tissues to obtain water and nutrients and incidentally to transfer genes, mRNA, herbivory-induced signals, and viruses (2, 6–9). How endophytes may influence interactions between plant parasites and their hosts is unknown.

Here, we report the results of 16S rRNA gene amplicon sequencing of bacterial endophytes from parasitized and unparasitized samples of the common sunflower (*Helianthus annuus*) and samples of its associated plant parasite field dodder (*Cuscuta campestris*) (Table 1).

**TABLE 1:** Sample information for sequencing reads (final length of 251 bp)

Sample^*a*^	Host species	Ecological status	Site on sunflower in reference to haustorium attachment	No. of raw sequencing reads	No. of quality-filtered reads	SRA accession no.
DodderB1	*Cuscuta campestris*	Parasite	NA^*b*^	6,309	3,775	SRR12442295
DodderB2	*Cuscuta campestris*	Parasite	NA	4,605	3,358	SRR12442294
DodderB3	*Cuscuta campestris*	Parasite	NA	5,973	3,763	SRR12442283
DodderB4	*Cuscuta campestris*	Parasite	NA	10,660	6,479	SRR12442272
DodderB5	*Cuscuta campestris*	Parasite	NA	4,580	2,291	SRR12442262
DodderB6	*Cuscuta campestris*	Parasite	NA	780	419	SRR12442261
DodderB7	*Cuscuta campestris*	Parasite	NA	6,369	4,421	SRR12442260
DodderB8	*Cuscuta campestris*	Parasite	NA	1,059	637	SRR12442259
InfectedAbove1	*Helianthus annuus*	Parasitized	Above attachment site	7,577	4,932	SRR12442258
InfectedAbove10	*Helianthus annuus*	Parasitized	Above attachment site	6,336	5,024	SRR12442257
InfectedAbove11	*Helianthus annuus*	Parasitized	Above attachment site	8,562	5,338	SRR12442293
InfectedAbove2	*Helianthus annuus*	Parasitized	Above attachment site	15,006	8,254	SRR12442292
InfectedAbove3	*Helianthus annuus*	Parasitized	Above attachment site	13,149	7,045	SRR12442291
InfectedAbove4	*Helianthus annuus*	Parasitized	Above attachment site	14,382	7793	SRR12442290
InfectedAbove6	*Helianthus annuus*	Parasitized	Above attachment site	21,060	11,832	SRR12442289
InfectedAbove7	*Helianthus annuus*	Parasitized	Above attachment site	9,685	5,773	SRR12442288
InfectedAbove8	*Helianthus annuus*	Parasitized	Above attachment site	7,644	5,098	SRR12442287
InfectedAbove9	*Helianthus annuus*	Parasitized	Above attachment site	9,195	5,537	SRR12442286
InfectedDown1	*Helianthus annuus*	Parasitized	At attachment site	209	87	SRR12442285
InfectedDown10	*Helianthus annuus*	Parasitized	At attachment site	22,916	13,033	SRR12442284
InfectedDown11	*Helianthus annuus*	Parasitized	At attachment site	287	129	SRR12442282
InfectedDown2	*Helianthus annuus*	Parasitized	At attachment site	420	168	SRR12442281
InfectedDown3	*Helianthus annuus*	Parasitized	At attachment site	287	134	SRR12442280
InfectedDown4	*Helianthus annuus*	Parasitized	At attachment site	246	117	SRR12442279
InfectedDown6	*Helianthus annuus*	Parasitized	At attachment site	395	182	SRR12442278
InfectedDown7	*Helianthus annuus*	Parasitized	At attachment site	333	174	SRR12442277
InfectedDown8	*Helianthus annuus*	Parasitized	At attachment site	971	517	SRR12442276
InfectedDown9	*Helianthus annuus*	Parasitized	At attachment site	20,661	11,716	SRR12442275
UninfectedDown1	*Helianthus annuus*	Unparasitized	At attachment site (if haustoria had been present)	23,047	18,520	SRR12442274
UninfectedDown10	*Helianthus annuus*	Unparasitized	At attachment site (if haustoria had been present)	14,840	6,883	SRR12442273
UninfectedDown11	*Helianthus annuus*	Unparasitized	At attachment site (if haustoria had been present)	25,432	16,125	SRR12442271
UninfectedDown12	*Helianthus annuus*	Unparasitized	At attachment site (if haustoria had been present)	10,675	5,634	SRR12442270
UninfectedDown13	*Helianthus annuus*	Unparasitized	At attachment site (if haustoria had been present)	12,185	6,183	SRR12442269
UninfectedDown2	*Helianthus annuus*	Unparasitized	At attachment site (if haustoria had been present)	23,781	19,594	SRR12442268
UninfectedDown3	*Helianthus annuus*	Unparasitized	At attachment site (if haustoria had been present)	19,725	14,828	SRR12442267
UninfectedDown4	*Helianthus annuus*	Unparasitized	At attachment site (if haustoria had been present)	29,750	22,466	SRR12442266
UninfectedDown6	*Helianthus annuus*	Unparasitized	At attachment site (if haustoria had been present)	17,491	8,438	SRR12442265
UninfectedDown7	*Helianthus annuus*	Unparasitized	At attachment site (if haustoria had been present)	8,759	4,875	SRR12442264
UninfectedDown8	*Helianthus annuus*	Unparasitized	At attachment site (if haustoria had been present)	10,046	5,408	SRR12442263

a All samples were collected from the north side of Mount Whitney Avenue in the town of Huron, Fresno County, California (36°25′49″N, 120°10′53″W), on 12 August 2017.

b NA, not applicable.

Stem tissues from randomly selected parasitized sunflower-dodder pairs (*n* = 15) and unparasitized sunflower plants (*n* = 15) were collected and transported on ice to the laboratory for surface sterilization (10). Briefly, 10 to 20 g of tissue was sterilized through two rounds of submersion in phosphate-buffered saline for 2 min, followed by 70% ethanol for 1 min and then 30% (round 1) or 3% (round 2) hydrogen peroxide (H_2_O_2_) for 3 min. Samples were then rinsed three times with deionized water. Sterilization was verified by plating onto 1/10 strength (4 g of Trypticase soy agar and 15 g of Bacto agar per liter) Trypticase soy agar plates, with incubation for 10 days at 30°C. DNA was extracted as described previously (10). We modified the published protocol by resuspending the air-dried pellet with 30 μl of sterile, deionized water.

We performed a nested PCR with the chloroplast-excluding primers 16S 799f (AACMGGATTAGATACCCKG) and 16S 1492r (TACGGHTACCTTGTTCGACTT) (11). The 50-μl PCR mixture contained 100 ng of genomic DNA, 1 μl of each primer at 10 nmol/liter, 10 μl of 5× GoTaq buffer, 5 μl of 25 mmol/liter MgCl_2_, 2.5 μl of 1 mg/ml bovine serum albumin, 1 μl of deoxynucleoside triphosphates (dNTPs), each at 10 mmol/liter, and 2.5 U GoTaq polymerase. Thermocycler conditions were initial denaturing for 3 min at 95°C, 20 cycles of 40 s at 95°C, 40 s at 50°C, and 90 s at 72°C and a final 10-min elongation at 72°C (12). Agarose (1%) gel electrophoresis was used to separate the PCR products. The bacterial product band (∼750 bp) was excised and purified using the Zymoclean gel DNA recovery kit (Zymo Research, Irvine, CA). The purified DNA was reamplified using the barcoded primer set 16S 799f and 16S 1115r (AGGGTTGCGCTCGTTG) (13), using the same conditions as described above. The resultant 300- to 400-bp band was excised and purified as described above. Sequencing using the two-step amplicon-to-data approach was performed by the Microbial Analysis, Resources, and Services facility at the University of Connecticut with an Illumina MiSeq system to generate 2 × 250-bp reads (14).

**FIG 1:**
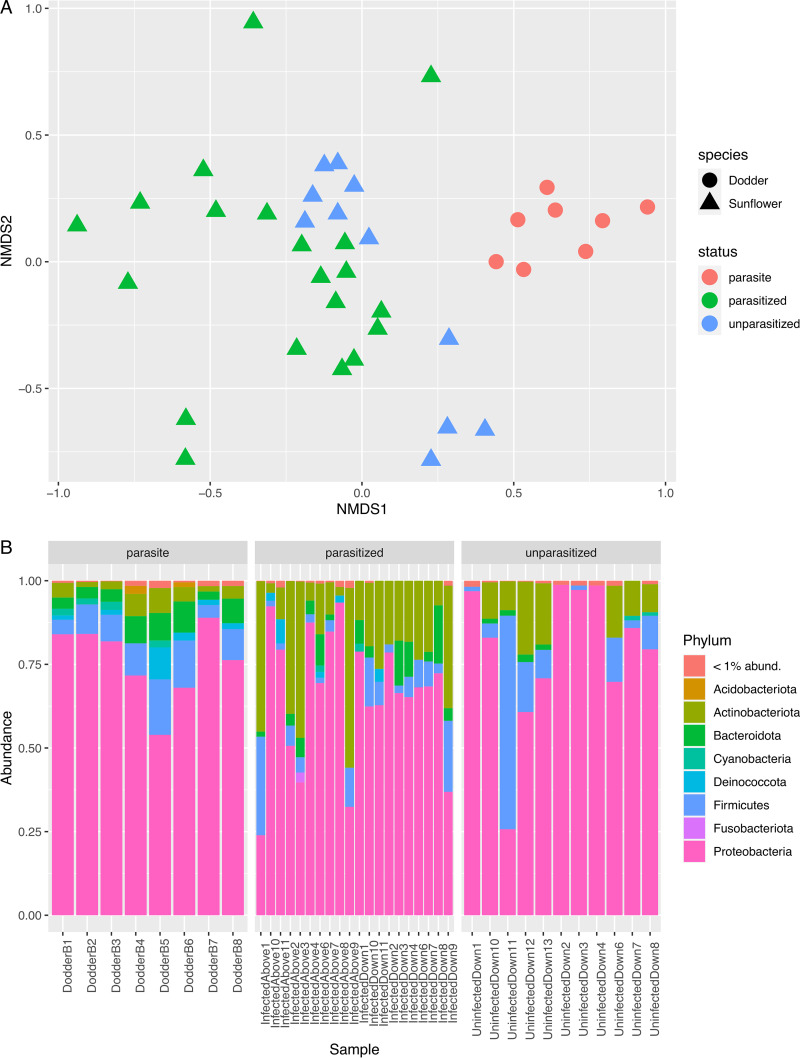
(A) Nonmetric multidimensional scaling (NMDS) based on Bray-Curtis distances. Shapes correspond to sample species, and colors correspond to sample status (parasite, parasitized, or unparasitized). (B) Relative abundance of phyla obtained from 16S rRNA sequencing of dodder (parasite), parasitized sunflower, and unparasitized sunflower samples. Phyla with a relative abundance of less than 1% and unassigned amplicon sequence variants were grouped together in their own category.

The paired-end demultiplexed sequences were imported using QIIME2 v2020.6 (15), and the DADA2 plugin (16) was used to denoise the sequences and to remove phiX and chimeric sequences. Based on the quality plot generated, 10 bp was trimmed from the beginning of each sequence and reads were truncated at 220 bp. The numbers of reads before and after use of the DADA2 pipeline are listed in Table 1. Taxonomy was assigned using the Silva v138 database (17–19). Data were exported using qiime2R v0.99.34 (https://github.com/jbisanz/qiime2R) for analysis with phyloseq v1.28.0 (20), vegan v2.5.6 (https://cran.r-project.org/package=vegan), and ggplot2 v3.3.2 (21). Using Bray-Curtis distances, there were significant differences between endophyte communities in parasitized and unparasitized sunflowers (permutational multivariate analysis of variance [PERMANOVA], *P *=* *0.001) and between endophyte communities in dodder and sunflowers (PERMANOVA, *P *=* *0.001) (Fig. 1A). The predominant phyla in all samples were *Proteobacteria*, *Firmicutes*, *Bacteroidota*, and *Actinobacteria* (Fig. 1B).

### Data availability.

The 16S rRNA gene amplicon sequence data have been deposited in the GenBank Sequence Read Archive (SRA) under the BioProject accession number PRJNA656591 (Table 1).
